# Maize ZmBES1/BZR1-3 and -9 Transcription Factors Negatively Regulate Drought Tolerance in Transgenic *Arabidopsis*

**DOI:** 10.3390/ijms23116025

**Published:** 2022-05-27

**Authors:** Wenqi Feng, Yuan Liu, Yang Cao, Yiran Zhao, Hongwanjun Zhang, Fuai Sun, Qingqing Yang, Wanchen Li, Yanli Lu, Xuecai Zhang, Fengling Fu, Haoqiang Yu

**Affiliations:** 1 Key Laboratory of Biology and Genetic Improvement of Maize in Southwest Region, Ministry of Agriculture, Maize Research Institute, Sichuan Agricultural University, Chengdu 611130, China; fwq@stu.sicau.edu.cn (W.F.); 18394159617@163.com (Y.L.); caoy@stu.sicau.edu.cn (Y.C.); zhaoyiran1210@163.com (Y.Z.); zhanghwj95@163.com (H.Z.); b20170405@stu.sicau.edu.cn (F.S.); yqingqing1992@163.com (Q.Y.); aumdyms@sicau.edu.cn (W.L.); luyanli@sicau.edu.cn (Y.L.); 2 International Maize and Wheat Improvement Center (CIMMYT), Texcoco 56237, Mexico; xc.zhang@cgiar.org

**Keywords:** maize, BES1/BZR1s, transcription factor, abiotic stress, drought

## Abstract

The BRI1-EMS suppressor 1 (BES1)/brassinazole-resistant 1(BZR1) transcription factors play crucial roles in plant growth, development, and stress response. However, little is known about the function of maize’s BES1/BZR1s. In this study, the *ZmBES1/BZR1-3* and *ZmBES1/BZR1-9* genes were cloned from maize’s inbred line, B73, and they were functionally evaluated by analyzing their expression pattern, subcellular localization, transcriptional activation activity, as well as their heterologous expression in *Arabidopsis*, respectively. The results of the qRT-PCR showed that the *ZmBES1/BZR1-3* and *ZmBES1/BZR1-9* genes were predominantly expressed in the root, and their expression was significantly down-regulated by drought stress. The ZmBES1/BZR1-3 and ZmBES1/BZR1-9 proteins localized in the nucleus but showed no transcriptional activation activity as a monomer. Subsequently, it was found that the heterologous expression of the *ZmBES1/BZR1-3* and *ZmBES1/BZR1-9* genes in *Arabidopsis* decreased drought tolerance, respectively. The transgenic lines showed a more serious wilting phenotype, shorter root length, lower fresh weight, and higher relative electrolyte leakage (REL) and malondialdehyde (MDA) content compared to the control under drought stress. The RNA-sequencing data showed that the 70.67% and 93.27% differentially expressed genes (DEGs) were significantly down-regulated in *ZmBES1/BZR1-3* and *ZmBES1/BZR1-9* transgenic *Arabidopsis*, respectively. The DEGs of *ZmBES1/BZR1-3* gene’s expressing lines were mainly associated with oxidative stress response and amino acid metabolic process and enriched in phenylpropanoid biosynthesis and protein processing in the endoplasmic reticulum. But the DEGs of the *ZmBES1/BZR1-9* gene’s expressing lines were predominantly annotated with water deprivation, extracellular stimuli, and jasmonic acid and enriched in phenylpropanoid biosynthesis and plant hormone signal transduction. Moreover, *ZmBES1/BZR1-9* increased stomatal aperture in transgenic *Arabidopsis* under drought stress. This study indicates that ZmBES1/BZR1-3 and ZmBES1/BZR1-9 negatively regulate drought tolerance via different pathways in transgenic *Arabidopsis*, and it provides insights into the underlying the function of BES1/BZR1s in crops.

## 1. Introduction

Drought stress is one of the major environmental stimuli with deleterious effects on plant growth and development [1]. To survive under these changes, plants have developed multifaceted strategies at the morphology, physiology, and photosynthesis levels during the evolution process [2,3,4,5]. Among these cues, the induction of various transcription factors (TFs) regulating the expression of downstream genes is one of the most important approaches to plant stress responses [6,7]. The BRI1-EMS1 suppressor (BES1)/brassinazole-resistant 1 (BZR1) TFs are a kind of plant-specific TF family [8,9,10]. BES1/BZR1s are activated by brassinosteroid (BR); then, they bind to the E-box (CANNTG) or BRRE (CGTGT/CG) element of the downstream gene’s promoter to regulate their transcription and transduce the BR signal [9,11,12,13].

Previous studies have found that BES1/BZR1s are involved in various biological and developmental processes and in the stress response of plants. For instance, BES1 promotes the transcription of BEE1 (BR ENHANCED EXPRESSION 1) to control the flowering of the blue light signaling pathway [14]. BZR1 also enhances photosynthesis through upregulating the transcription of Calvin cycle genes, including *FBA1*, *RCA1*, *FBP5*, and *PGK1* [15]. BZR1 and BES1 are antagonized by C3H15 to regulate the expression of the cell elongation-associated target gene, *SMALL AUXIN-UP RNA 15* (*SAUR15*), to control cell elongation [16]. Likewise, BZR1 directly binds to the promoter of the *HPPD* (4-HYDROXYPHENYLPYRUVATE DIOXYGENASE) gene to suppress its expression, resulting in decreasing carotenoid production [17]. In addition, BES1/BZR1s interact with other proteins to regulate the transcription of downstream genes. The interaction of BZR1 and ARR (ARABIDOPSIS RESPONSE REGU LATORs) positively promotes ovule initiation and seed production [18]. BZR1 interacts with SPL9 to coordinately regulate the downstream genes’ expression to regulate the vegetative phase change and cell elongation [19]. In *Arabidopsis*, BES1 interacts with drought-induced RD26 and WRKY54 TFs to negatively regulate its drought response [20,21]; but TaBZR2 and ZmBES1/BZR1-5, the BES1/BZR1 family member from wheat and maize, positively regulate drought response by activating downstream genes, respectively [22,23]. Besides, the BES1/BZR1 family members promote heat and freezing tolerance via directly regulating the expression of *HSF* (heat shock factor), *CBF* (C-repeat binding factor), and *COR* (cold-regulated) genes, respectively [24,25]. However, the function of BES1/BZR1 in crops remains obscure.

In previous studies, it has been demonstrated that there are eleven ZmBES1/BZR1 TFs in the maize genome [26,27]. However, only ZmBES1/BZR1-2 (Zm00001d039439) and ZmBES1/BZR1-5 (Zm00001d053975) are functionally validated in regulating seed size and stress response [23,28,29]. The function of other *ZmBES1/BZR1* genes remains unknown. In this study, the *ZmBES1/BZR1-3* and *ZmBES1/BZR1-9* genes were cloned, and their function was evaluated by analyzing their expression pattern under drought stress, subcellular localization, transcriptional activation activity, as well as the heterologous expression in transgenic *Arabidopsis*.

## 2. Results

### 2.1. Expression Patterns of ZmBES1/BZR1-3 and ZmBES1/BZR1-9 in Maize

**Figure 1:**
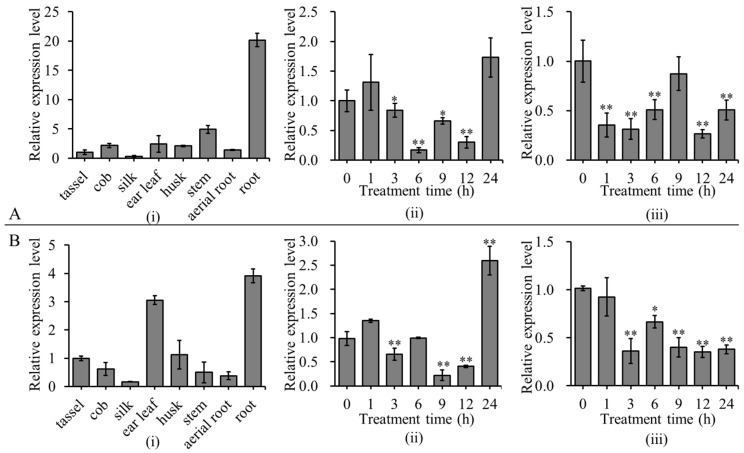
Expression patterns of *ZmBES1/BZR1-3* (**A**) and *ZmBES1/BZR1-9* (**B**). (**i**) Expression pattern in different tissues of maize. (**ii**); (**iii**) expression pattern in the shoot and root under drought stress, respectively. The three-leaf old seedlings of maize’s inbred line, B73, were subjected to 16% PEG-6000 to mimic drought stress. The relative expression level was calculated and normalized using the 2^−ΔΔCT^ method of the CFX Manger™ software version 2.0 (Bio-Rad, Berkeley, CA, USA). * and ** represent *p* < 0.05 and *p* < 0.01 by the Student’s *t*-test, respectively.

To analyze the tissue-specific expression and stress-responsive expression of *ZmBES1/BZR1-3* and *ZmBES1*/*BZR1-9*, a quantitative real-time PCR (qRT-PCR) was performed. As shown in Figure 1, the *ZmBES1/BZR1-3* and *ZmBES1/BZR1-9* genes were predominantly expressed in maize roots; likewise, they were highly expressed in leaves. In addition, the expression of *ZmBES1/BZR1-3* and *ZmBES1/BZR1-9* genes under drought stress showed a similar pattern. Their expression in the shoot and root of seedlings was both significantly inhibited by drought stress. The expression of *ZmBES1/BZR1-3* reached the lowest level in the shoot and root at 6 h and 12 h of treatment, respectively. The expression of *ZmBES1/BZR1-9* reached the lowest level in the shoot and root at 9 h and 12 h of treatment, respectively. However, the expression of *ZmBES1/BZR1-9* was significantly up-regulated at 24 h of treatment. The result suggests that *ZmBES1/BZR1-3* and *ZmBES1/BZR1-9* might function in regulating drought tolerance.

### 2.2. ZmBES1/BZR1-3 and ZmBES1/BZR1-9 Localized in the Nucleus

**Figure 2:**
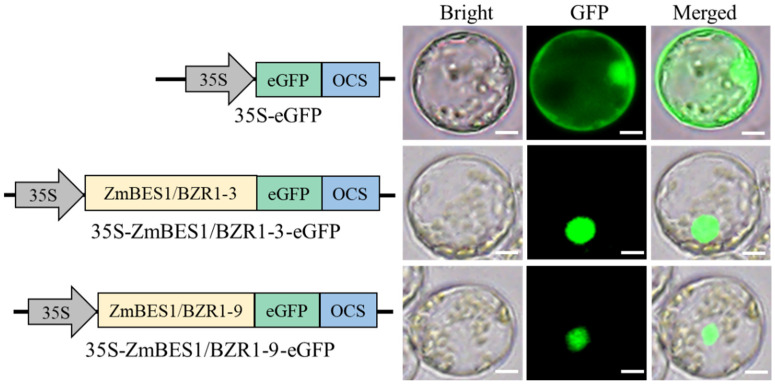
Subcellular localization. The ORF of *ZmBES1/BZR1-3* and *ZmBES1/BZR1-9*, without a stop codon, was fused to *eGFP* and transiently expressed in maize’s mesophyll protoplasts for 12 h. The scale bar was 20 μm.

BES1/BZR1 TF accumulates in the nucleus to regulate the downstream gene’s expression. Therefore, the open reading frame (ORF) of *ZmBES1/BZR1-3* and *ZmBES1/BZR1-9*, without a stop codon, was amplified and inserted into pC2300-*35S*-*eGFP* fused to *eGFP* and used to transient the expression in maize’s mesophyll protoplasts. The result exhibited that the green fluorescence (GFP) was observed in the whole cell, including the cytoplasm and nucleus of protoplasts transformed by *35S*-*eGFP’s* empty vector. However, the GFP was only observed in the nucleus of protoplasts transformed by *35S-ZmBES1/BZR1-3-eGFP* and *35S-ZmBES1/BZR1-9-eGFP*, respectively (Figure 2).

### 2.3. ZmBES1/BZR1-3 and ZmBES1/BZR1-9 Showed no Transcriptional Activation Activity in Yeast

**Figure 3:**
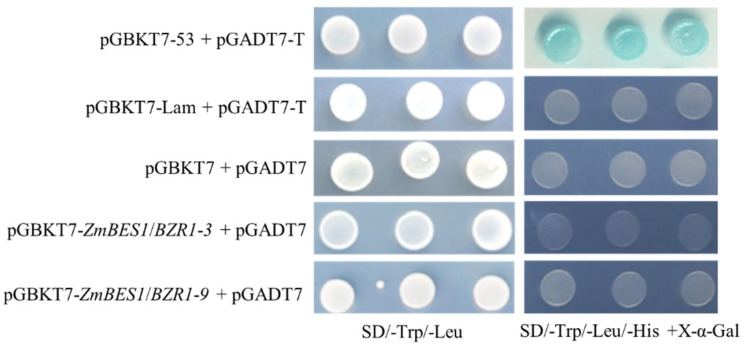
The transcriptional activation assay. The pGBKT7-*ZmBES1/BZR1-3* with pGADT7, and pGBKT7-*ZmBES1/BZR1-9* with pGADT7, were co-transformed into yeast, respectively. The co-transformation of pGBKT7-53 with pGADT7-T was used as the positive control. The co-transformation pGBKT7-Lam with pGADT7-T and pGBKT7 with pGADT7 were used as the negative control.

To verify the transcriptional activation activity of ZmBES1/BZR1-3 and ZmBES1/BZR1-9 proteins, the ORF of *ZmBES1/BZR1-3* and *ZmBES1/BZR1-9* was cloned, inserted into pGBKT7, and transformed into yeast AH109 with pGADT7, respectively. The results exhibited that the yeast strains, co-transformed by pGBKT7-*ZmBES1/BZR1-3* with pGADT7, pGBKT7-*ZmBES1/BZR1-9* with pGADT7, as well as the positive control (pGBKT7-53 with pGADT7-T) and negative control (pGBKT7-Lam with pGADT7-T, and pGBKT7 with pGADT7), grew normally and produced clones on SD/-Leu/-Trp plates, respectively. However, only the yeast strains that co-transformed pGBKT7-53 with pGADT7-T (positive control) could grow and turn blue on SD/-Leu/-Trp/-His with X-α-Gal plates. The yeast strains co-transformed by pGBKT7-*ZmBES1/BZR1-3* with pGADT7, pGBKT7-*ZmBES1/BZR1-9* with pGADT7, as well as the negative control could not grow on SD/-Leu/-Trp/-His with X-α-Gal plates (Figure 3). This finding suggests that ZmBES1/BZR1-3 and ZmBES1/BZR1-9 have no transcriptional activation activity as a monomer in yeast.

### 2.4. ZmBES1/BZR1-3 and ZmBES1/BZR1-9 Negatively Regulated Drought Tolerance in Arabidopsis

**Figure 4:**
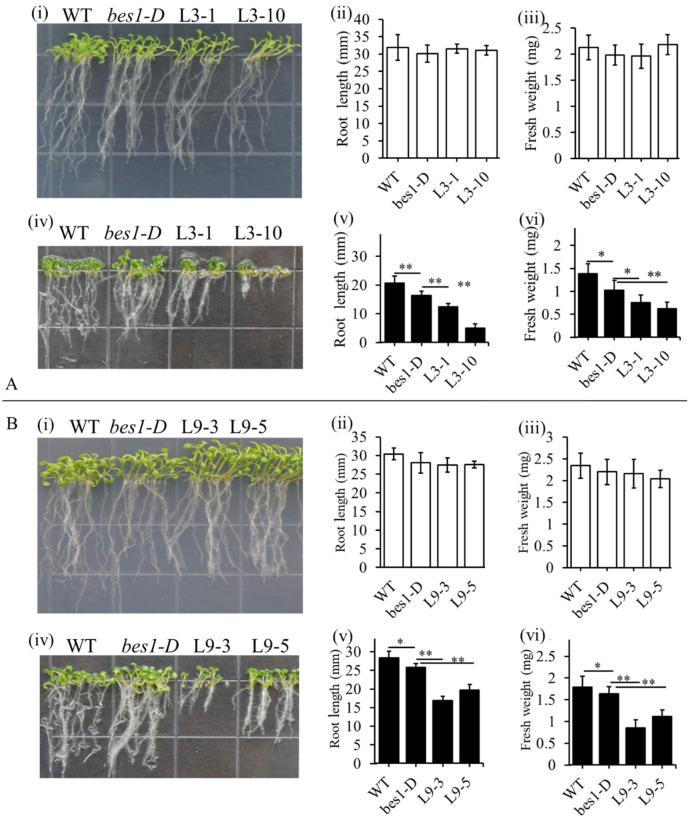
The phenotype of transgenic plants under drought stress mimicking by mannitol. (**A**) Phenotype of *ZmBES1/BZR1-3* expressing lines. (**B**) The phenotype of the *ZmBES1/BZR1-9* expressing lines. (**i**), (**ii**), (**iii**) The phenotype, root length, and fresh weight of every line on 1/2 MS plates, respectively. (**iv**), (**v**), (**vi**) The phenotype, root length, and fresh weight of every line on 1/2 MS plates with mannitol, respectively. The seeds of every line were surfaced-sterilized, planted on 1/2 MS plates without (control) or with mannitol, and vertically cultured for three weeks. All data are means (±SD) of three biological replicates. L3-1 and L3-10 are homozygous lines expressing *ZmBES1/BZR1-3*. L9-3 and L9-5 are homozygous lines expressing *ZmBES1/BZR1-9*. *bes1-D*, untransformed mutant. WT, wild type. * and ** represents *p* < 0.05 and *p* < 0.01, respectively.

To investigate the function of *ZmBES1/BZR1-3* and *ZmBES1/BZR1-9* in the drought stress response, they were transformed into *bes1-D*, the *Arabidopsis AtBES1* mutant, for phenotyping under the stress treatment. As shown in Figure 4, all transgenic lines expressing *ZmBES1/BZR1-3* (L3-1 and L3-10) or *ZmBES1/BZR1-9* (L9-3 and L9-5) showed no difference compared to *bes1-D* and the wild type (WT) on the 1/2 MS plates. However, under drought stress mimicked by a mannitol treatment, the transgenic lines L3-1, L3-10, L9-3, and L9-5 were much more sensitive to drought stress compared to the *bes1-D* seedlings that were more sensitive than WT, although all of the plants were inhibited by stress. The L3-1 and L3-10 lines showed significant differences compared to *bes1-D* under a 200 mM mannitol treatment, while L9-3 and L9-5 exhibited differences compared to *bes1-D* under a 150 mM mannitol treatment. The root length and fresh weight of the L3-1, L3-10, L9-3, and L9-5 lines were both significantly lower than *bes1-D* on the 1/2 MS plates with mannitol. Meanwhile, the root length and fresh weight of *bes1-D* were significantly lower than the WT.

**Figure 5:**
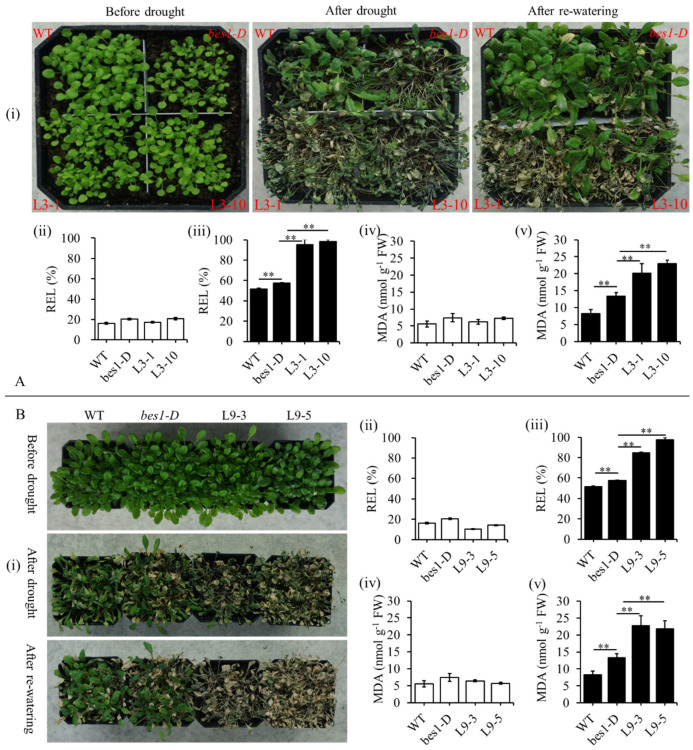
The phenotype of transgenic plants under the drought treatment. (**A**) The phenotype of the *ZmBES1/BZR1-3* expressing lines. (**B**) The phenotype of the *ZmBES1/BZR1-9* expressing lines. (**i**) The phenotype, (**ii**), (**iv**) the REL and MDA content before treatment, respectively. (**iii**), (**v**) The REL and MAD content after treatment, respectively. The seeds of every line were planted on the soil, cultured in the chamber, and irrigated at 4-day intervals. The 20-day-old seedlings were used for the drought treatment by holding water and subsequently monitored for the stress symptoms for three weeks. All of the values are means (±SD) of three biological replicates. L3-1, L3-10, L9-3, and L9-5 are transgenic lines; *bes1-D*, mutant. WT, wild type. ** represents *p* < 0.05 and *p* < 0.01, respectively.

Subsequently, the seeds of the L3-1, L3-10, L9-3, L9-5, *bes1-D*, and WT were sown into the soil for drought treatment. As shown in Figure 5, before the treatment, all of the plants grew well and showed a vigorous phenotype. The relative electrolyte leakage (REL) and malondialdehyde (MDA) content of all of the plants kept a low level and showed no significant difference. Whereas, after three weeks of drought stress without watering, the L3-1, L3-10, L9-3, and L9-5 lines showed serious a wilting phenotype, but *bes1-D* and WT showed slighter and greener phenotype compared to the transgenic lines. The REL and MDA content of the L3-1, L3-10, L9-3, and L9-5 lines was significantly higher than *bes1-D* and the WT, although all of them were increased after the drought treatment. Meanwhile, the *bes1-D* was more sensitive to drought stress than the WT.

The above results indicate that the expression of the *ZmBES1/BZR1-3* and *ZmBES1/BZR1-9* genes both decrease the drought tolerance in transgenic *Arabidopsis*.

### 2.5. ZmBES1/BZR1-3 and ZmBES1/BZR1-9 Inhibited Transcription of Stress-Related Genes in Transgenic Arabidopsis

**Figure 6:**
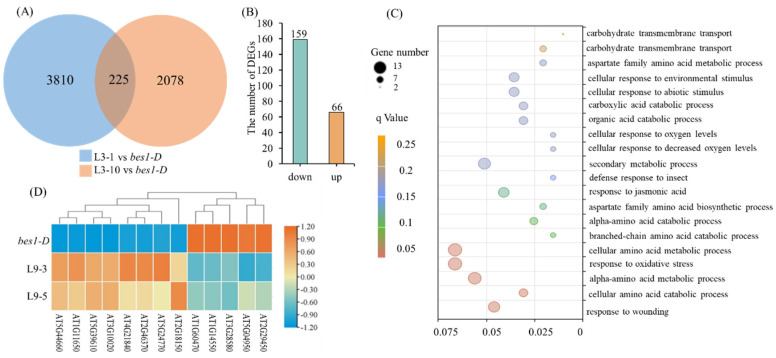
DEGs of transgenic *Arabidopsis* with the *ZmBES1/BZR1-3* gene compared to *bes1-D*. (**A**) DEGs of L3-1 and L3-10 compared to *bes1-D*, respectively. (**B**) The common DEGs are shared by L3-1 and L3-10. (**C**) GO analysis of common DEGs. (**D**) The heat map of the 13 DEGs responds to oxidative stress.

To explore the potential mechanism of ZmBES1/BZR1-3 and ZmBES1/BZR1-9 negatively regulating the drought tolerance, the differentially expressed genes (DEGs) in the transgenic lines, compared to *bes1-D*, were unraveled by the RNA-sequencing (RNA-seq). In transgenic *Arabidopsis* with the *ZmBES1/BZR1-3* gene, there were 225 common DEGs shared by the L3-1 and L3-10 lines (Figure 6A and Table S1 in the Supplementary Materials). Among these common DEGs, 70.67% DEGs (159) were down-regulated, and 29.33% DEGs (66) were up-regulated, respectively (Figure 6B). Gene Ontology (GO) analysis exhibited that the common DEGs were mainly enriched in the oxidative stress response and amino acid metabolic process (Figure 6C and Table S2). In addition, the KEGG enrichment analysis indicated that these common DEGs participated in phenylpropanoid biosynthesis and protein processing in the endoplasmic reticulum (Figure S1).

**Figure 7:**
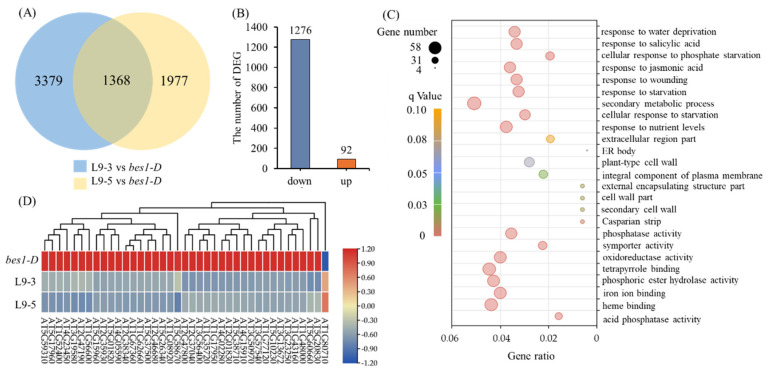
DEGs of transgenic *Arabidopsis* with the *ZmBES1/BZR1-9* gene compared to *bes1-D*. (**A**) DEGs of L9-3 and L9-5 compared to *bes1-D*, respectively. (**B**) The common DEGs are shared by L9-3 and L9-5. (**C**) GO analysis of common DEGs. (**D**) The heat map of the 39 DEGs responds to water deprivation.

In transgenic *Arabidopsis* with the *ZmBES1/BZR1-9* gene, there were 1368 common DEGs shared by the L9-3 and L9-5 lines (Figure 7A and Table S3). Among these DEGs, 93.27% of the DEGs (1276) were down-regulated, and only 6.73% of the DEGs (92) were up-regulated compared to *bes1-D*, respectively (Figure 7B). Furthermore, the result of the GO analysis showed that 39, 43, and 41 DEGs were annotated with response to water deprivation, extracellular stimuli, and jasmonic acid, respectively (Figure 7C and Table S4). Among the 39 DEGs that respond to water deprivation, 38 DEGs were down-regulated, and only one DEG was up-regulated (Figure 7D). Specifically, 12 down-regulated DEGs were well confirmed to positively regulate drought stress, including four genes (AT2G46680, AT2G47800, AT4G23450, and AT5G37500) positively regulate stomatal aperture, and one up-regulated DEG (AT1G80710) negatively regulates drought stress (Table S3). KEGG enrichment analysis showed that the DEGs were mainly enriched in phenylpropanoid biosynthesis and the plant hormone signal transduction pathways (Figure S2).

### 2.6. ZmBES1/BZR1-9 Increased Stomatal Aperture

**Figure 8:**
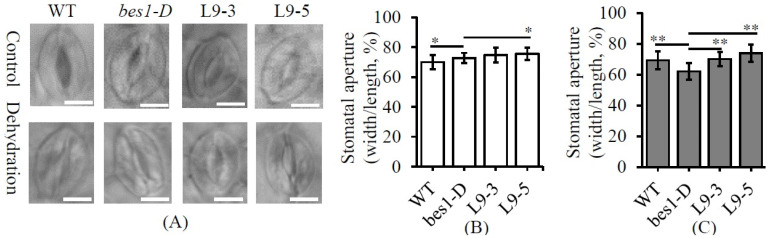
The stomatal phenotype. (**A**) The stomatal morphology. (**B**) The stomatal aperture before dehydration. (**C**) The stomatal aperture after 1 h of dehydration. The leaf of every line was detached for 1 h of dehydration treatment and used to measure the stomatal aperture. All values are means (±SD) of three biological replicates. L9-3 and L9-5 are transgenic lines. *bes1-D*, mutant. WT, wild type. * and ** represents *p* < 0.05 and *p* < 0.01, respectively. The scale bar was 10 μm.

Due to the four stomatal development genes, including AT4G23450 (*AtAIRP1*), AT4G23450 (*AtMRP4*), AT2G46680 (*AtHB7*), and AT5G37500 (*AtGORK*), were significantly down-regulated in the *ZmBES1/BZR1-9* transgenic lines, the stomatal phenotype of L9-3 and L9-5 was monitored. As shown in Figure 8, under an optimal condition, the stomata exhibited an opened status, and the stomatal aperture of L9-5 was significantly larger than *bes1-D*. However, after 1 h of dehydration, most of the stomata of L9-3 and L9-5 were kept open. The stomatal aperture of L9-3 and L9-5 was greatly bigger than that of *bes1-D*, respectively. Likewise, the stomatal aperture of *bes1-D* was greatly smaller than the WT. These results suggest that enhanced stomatal aperture was one cause of drought sensitivity in the L9-3 and L9-5 plants.

## 3. Discussion

The BES1/BZR1 family has been identified as the TF in several plant species and accumulates in the nucleus to activate or inhibit the downstream genes’ expression [26,30,31,32,33]. In the present study, we found that the ZmBES1/BZR1-3 and ZmBES1/BZR1-9 proteins localized in the nucleus and had no transcriptional activation activity (Figure 2 and Figure 3), which implies that ZmBES1/BZR1-3 and ZmBES1/BZR1-9 may function as TFs to regulate the downstream genes via requiring co-factors. Previous studies show that BES1/BZR1 interacts with other TFs to co-regulate their target genes’ expression [19,20,21,22]. Likewise, in our previous study, ZmBES1/BZR1-5 was also found to have no transcriptional activation as a monomer but functioned via homodimerization [28].

The gene expression patterns can provide information about gene function [34]. We found that the expression of *ZmBES1/BZR1-3* and *ZmBES1/BZR1-9* in maize was significantly inhibited by drought stress, although *ZmBES1/BZR1-9* was up-regulated at 24 h of treatment (Figure 1). It suggests that the ZmBES1/BZR1-3 and ZmBES1/BZR1-9 function in drought tolerance. Because the ZmBES1/BZR1-3 and ZmBES1/BZR1-9 shared a highly conserved bHLH domain, the characterised domain of BES1/BZR1, with *Arabidopsis* BES1/BZR1s (Supplementary Figure S3) [9,23,26,28]; hence, they were heterologously expressed in the *bes1-D* mutant. After the evaluation of the drought tolerance of transgenic *Arabidopsis*, it’s found that the heterologous expression of *ZmBES1/BZR1-3* and *ZmBES1/BZR1-9* in the *bes1-D* mutant both increase drought sensitivity of the transgenic lines (Figure 5). The *bes1-D* mutant also exhibited more sensitivity to drought stress than the WT in the study (Figure 4 and Figure 5). It was previously reported that *bes1-D* negatively regulates drought tolerance [20]. However, TaBZR2 and ZmBES1/BZR1-5 were reported to regulate the drought tolerance positively [22,23]. These findings mean that the BES1/BZR1 family members evolve a functional diversity and play dual roles in drought response. In addition, *ZmBES1/BZR1-3* and *ZmBES1/BZR1-9* were predominantly expressed in maize roots (Figure 1). The transgenic lines, likewise, exhibited shorter roots after mimicking drought stress (Figure 4). This concluded that ZmBES1/BZR1-3 and ZmBES1/BZR1-9 suppressed the root development, which resulted in an increase in drought sensitivity. Adjusting the root system architecture is one important clue for plants to conquer stress due to the root being the first defensive line to adapt to their environment [35,36]. Moreover, *bes1-D* had a shorter meristem size in the root [37]. BES1 regulates the vascular cell development in the root apex and root growth mediated by the type one protein phosphatases [37,38]. Besides, BES1 acts to promote seedling growth in the auxin pathway [39].

Although ZmBES1/BZR1-3 and ZmBES1/BZR1-9 showed similar expression patterns (Figure 1), ZmBES1/BZR1-3 regulated the expression of the oxidative-stress responsive genes (Figure 6), while ZmBES1/BZR1-9 inhibited the water response genes’ expression (Figure 7), suggesting that they act on different signaling. These DEGs were enriched in phenylpropanoid biosynthesis, a protein processing in the ER, and hormone signal transduction (Figures S1 and S2), which showed positive roles in the drought stress response [5,40,41]. Among the 13 oxidative-stress responsive DEGs of the *ZmBES1/BZR1-3* expressing lines, the up-regulated gene, AT5G39610 (*ORE1* or *NAC092*), negatively regulates oxidative stress [42,43], and the down-regulated gene AT1G60470 (Galactinol synthase, *GolS*) positively improves the drought tolerance [44,45]. Moreover, the transgenic lines with the *ZmBES1/BZR1-9* gene also showed the stomatal insensitivity to drought compared to *bes1-D*, while the stomatal aperture of the *bes1-D* plants was smaller than the WT under stress (Figure 8). It could be explained that AtBES1 regulated drought tolerance via acting on the RD26 and WRKY TFs [21,22]. However, ZmBES1/BZR1-9 inhibited the expression of AT4G23450 (*AtAIRP1*) and AT2G47800 (*AtMRP4*), and the AT2G46680 (*AtHB7*) and AT5G37500 (*AtGORK*) genes (Figure 7 and Table S2), which has been proved to positively regulate drought tolerance via a coordinating stomatal movement [46,47]. Previous studies confirmed that many genes in the BR signaling pathway played a positive role in controlling the stomatal open [48]. Recently, BZR1 was found to promote the sugar-promoted expression of *β-AMYLASE1* (*BAM1*), which was responsible for starch degradation in the guard cells and increased the effects of BR on the stomatal opening [49,50].

**Figure 9:**
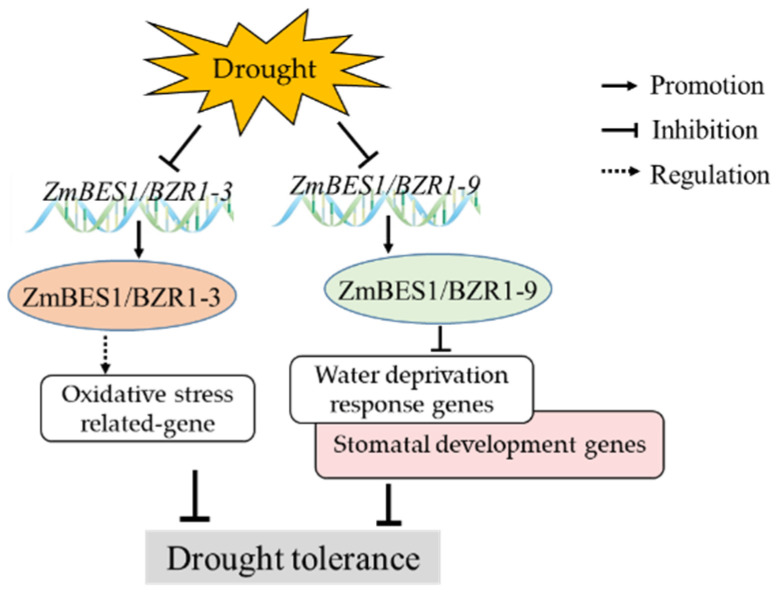
Diagram of the ZmBES1/BZR1-3 and ZmBES1/BZR1-9 regulated the network in the drought response.

In conclusion, the *ZmBES1/BZR1-3* and *ZmBES1/BZR1-9* genes were cloned from maize and confirmed to be inhibited by drought stress in the present study. The ZmBES1/BZR1-3 and ZmBES1/BZR1-9 proteins both localized in the nucleus and showed no transcriptional activation activity as the monomer. However, the heterologous expression of the *ZmBES1/BZR1-3* and *ZmBES1/BZR1-9* genes negatively regulate drought tolerance in *Arabidopsis* via different pathways (Figure 9).

## 4. Materials and Methods

### 4.1. Plant Materials and Growth Conditions

The seeds of the maize’s inbred line, B73, were germinated in filter paper, then transplanted into a Hoagland’s solution for a hydroponic culture under 16 h of light at 28 °C/8 h and in the dark at 25 °C. As described by Sun et al. [23], at the three-leaf old stage, the seedlings of the same size were subjected to 16% PEG-6000 to mimic drought stress. At 0, 1, 3, 6, 9, 12, and 24 h of treatment, the shoot and root were sampled, frozen in liquid nitrogen, and stocked at −80 °C for an RNA isolation. Meanwhile, the tassel, cob, silk, ear leaf, husk, stem, aerial root, and root of B73 were sampled for an RNA extraction, respectively. The seeds of the *Arabidopsis BES1* gene mutant (*bes1-D*, CS65988) were purchased from The *Arabidopsis* Information Resource (TAIR, https://www.arabidopsis.org/, accessed on 8 August 2019) and grown in a greenhouse at 22 °C and 60–70% relative humidity under a 10 h light/14 h dark photoperiod.

### 4.2. Expression Analysis of the ZmBES1/BZR1-3 and ZmBES1/BZR1-9 Gene

The total RNA was extracted from the above samples by an RNAiso plus kit (TaKaRa, Dalian, China), removed genomic DNA by digesting with RNAse-free DNAse (TaKaRa, Dalian, China), quantified using NanoDrop^TM^ One^C^ (ThermoScientific, Waltham, MA, USA), and reversely transcribed into cDNA according to the PrimeScript^TM^ reagent kit (TaKaRa, Dalian, China) according to the manufacturer’s instruction, respectively. The specific primer pairs of D3-F (5′-CGCTATCGTGTGGAACCTGA-3′) with D3-R (5′-CACGAGACCGTAACTCCGTC-3′), and D9-F (5′-GCTTACAACGGCCTCAGCTA-3′) with D9-R (5ʹ-GTAGCGGG-ACAGGTTGTTGA-3′), were designed using the Primer-BLAST at the NCBI website (https://www.ncbi.nlm.nih.gov/tools/primer-blast/index.cgi?LINK_LOC=BlastHome, accessed on 28 March 2020), synthesized at Sangon Biotech (Chendu, China), and used to amplify the 98 and 192 bp of the *ZmBES1/BZR1-3* and *ZmBES1/BZR1-9* genes, respectively. A pair of primers, TUB-F (5′-CTACCTCACGGCATCTGCTATGT-3′) and TUB-R (5′-GTCACACACACTCGACTTCACG-3′), were designed, synthesized, and used to amplify the 139 bp of the *ZmTUB* gene, which was used as an internal reference. According to the methods of Sun et al. [23], the qRT-PCR was performed in the Bio-Rad CFX96^TM^ Real-Time PCR system with a TransScript^®^ II Two-Step RT-PCR SuperMix (Transgen, Beijing, China) according to the manufacturer’s instructions.

### 4.3. Subcellular Localization

The ORF sequence without a stop codon of *ZmBES1/BZR1-3* and *ZmBES1/BZR1-9* was cloned from a cDNA sample using the primers PC-3F (5′-CGGGATCCATGGAGGGAGGGGTAGGAGCGGGAGGAAG-3′; the underlined sequences are the *Bam*H I sites) with PC-3R (5′-ACGCGTCGACCGAAAACCTTTCTTGCTGCTCCCTCATAG-3′; the underlined sequences are the *Sal* I sites), and PC-9F (5′-TCCCCCGGGATGAACGGGGGAGAAGGAGAAGGAG-3′ the underlined sequences are the *Sma* I sites) with PC-9R (5′-GGACTAGTAGCGCGGTCAGCGCCGG-3′; the underlined sequences are the *Spe* I sites), respectively. The PCR products and pC2300-*35S*-*eGFP* plasmids were digested using *Bam*H I/*Sal* I and *Sma* I/*Spe* I, respectively. Then, the ORF of *ZmBES1/BZR1-3* and *ZmBES1/BZR1-9* was inserted into the *Bam*H I/*Sal* I sites, and the *Sma* I/*Spe* I sites of pC2300-*35S*-*eGFP* to generate *35S-ZmBES1/BZR1-3-eGFP* and *35S-ZmBES1/BZR1-9-eGFP*, respectively. According to the methods described by Fu et al. [51], the 20 g leaves from the etiolated seedlings were cut into strips and digested into a 50 mL tube containing a 10 mL enzyme solution with 1.5% cellulase R10 (Yakult, Japan), 0.3% macerozyme R10 (Yakult, Japan), 0.6 M D-mannitol (Sigma, MO, USA), 10 mM MES (pH 5.7) (Sigma), 1 mM CaCl_2_ (Sigma), and 0.1% BSA (Sigma) for 6 h at 28 °C. The protoplasts were collected by centrifugating at 100× *g* and resuspended using a W5 solution (pH 5.7) with 154 mM NaCl (Sigma), 125 mM CaCl_2_ (Sigma), 5 mM KCl (Sigma), and 2 mM MES (Sigma). Then, the maize’s mesophyll protoplasts were used to transiently express the ZmBES1/BZR1-3 and ZmBES1/BZR1-9 proteins. The *35S-ZmBES1/BZR1-3-eGFP*, *35S-ZmBES1/BZR1-9*-*eGFP*, and *35S-eGFP* plasmid (control) were transformed into the 100 μL protoplasts, respectively. Subsequently, the protoplasts were incubated at 25 °C in the dark for 12 h, sampled, and used for GFP fluorescence imaging using the confocal microscope LSM800 (Carl Zeiss, Oberkochen, Germany).

### 4.4. Transcriptional Activation Activity Assay in Yeast

The transcriptional activation activity assay was performed as described by Lu et al. [52]; the ORF sequence of *ZmBES1/BZR1-3* and *ZmBES1/BZR1-9* was cloned from a cDNA sample using primers BD-3F (5′-AGAATTCATGGAGGGAGGGGTAGGAG-3′; the underlined sequences are the *Eco*R I sites) with BD-3R (5′-CGGGATCCTCACGAAAACCTTTCTTGCTGC-3′; the underlined sequences are the *Bam*H I sites), and BD-9F (5′-TTACAATCATATGATGAACGGGGGAGAAGGA-3′; the underlined sequences are the *Nde* I sites) with BD-9R (5′-TGAATTCTCAAGCGCGGTCAGC-3ʹ; the underlined sequences are the *Eco*R I sites), respectively. The PCR products and pGBKT7 plasmids were digested using *Eco*R I/*Bam*H I and *Nde* I/*Eco*R I, respectively. Then, the ORF of *ZmBES1/BZR1-3* and *ZmBES1/BZR1-9* was inserted into the *Eco*R I/*Bam*H I and *Nde* I/*Eco*R I sites of pGBKT7 to generate pGBKT7*-ZmBES1/BZR1-3* and pGBKT7*-ZmBES1/BZR1-9*, respectively. The pGBKT7-*ZmBES1/BZR1-3* and pGBKT7-*ZmBES1/BZR1-9* with the combination of pGADT7 were co-transformed into the yeast strain, AH109, respectively, and were cultured on SD/-Leu/-Trp and SD/-Leu/-Trp/-His/+X-α-Gal. The co-transformation of pGBKT7-53 and pGADT7-T was used as the positive control. The co-transformation of pGBKT7-Lam and pGADT7-T, as well as pGBKT7 and pGADT7, was used as the negative control. The yeast strains were cultured 3 days at 28 °C and used for phenotyping.

### 4.5. Plant Transformation and Phenotyping

The above constructs of the *35S-ZmBES1/BZR1-3* and *35S-ZmBES1/BZR1-9* plasmids were transformed into the *Agrobacterium tumefaciens* strain GV3101. Subsequently, the *Arabidopsis* plants (*bes1-D* mutant, CS65988) were transformed by the GV3101 strains with *ZmBES1/BZR1-3* and *ZmBES1/BZR1-9* through the floral-dipping method, respectively. The seeds of the T_0_ plants were collected, surface-sterilized using 75% ethanol for 1 min and in 5% NaClO for 5 min, washed three times with sterile distilled water, then planted on 1/2 MS plates with 50 mg/L kanamycin (Sigma, Saint Louis, MO, USA) for screening of positive transformants. The homozygous lines without the segregation of kanamycin-resistance in T_3_ were screened using the same method. The homozygous lines L3-1 and L3-10 of *ZmBES1/BZR1-3* expressing *Arabidopsis*, L9-3, and L9-5 of *ZmBES1/BZR1-3* expressing *Arabidopsis* were selected for the next studies.

The seeds of the homozygous lines L3-1, L3-10, L9-3, and L9-5, and the WT and *bes1-D* were surfaced-sterilized, sown on 1/2 MS plates with 0, 150, and 200 mM mannitol and vertically cultured for three weeks. Then the phenotype was photographed. The root length and fresh weight of every line were measured, respectively. Meanwhile, the seeds of every line were grown in soil in a growth chamber and irrigated at 4-day intervals. The 20-day-old seedlings were divided into four groups. Three of them were used for drought treatment by holding water and subsequently monitored for stress symptoms. Two weeks later, two groups of seedlings treated by drought with one group of seedlings under optimal conditions were used to determine the REL and MDA content according to the methods of Sun et al. [23] and Yu et al. [53], respectively. Another group of seedlings was treated for three weeks, then re-watered with a recovery time of three days and used to photograph wilting phenotype.

### 4.6. RNA-Seq Analysis

For the RNA-seq analysis, the total RNA was extracted from the 14-day-old seedlings of *bes1-D*, L3-1, L3-10, L9-3, and L9-10 using an RNAprep Pure Plant Kit (Tiangen, Beijing, China), respectively. The RNA’s integrity was evaluated by Bioanalyzer 2100 (Agilent, CA, USA) (RIN ≥ 7, 28S/18S ≥ 1.5). According to the methods of Sun et al. [23], the RNA-seq library was constructed using a VAHTSTM mRNA-seq V2 Library Prep Kit and sequenced using a novaseq6000 system at the Beijing Novogene Technology Company (Beijing, China). The sequencing data were evaluated, filtered, mapped to the *Arabidopsis* genome (TAIR 10), and used for assembling the transcripts and calculating the gene expression using FastQC (version 0.11.2), Trimmomatic (version 0.36) hisat2, and StringTie, respectively [54,55]. The DEGs were analyzed using the DESeq2 R package (1.20.0) with a *p*-value < 0.05 and |FoldChange| > 1. GO, and the KEGG enrichment analysis of the DEGs was implemented by the clusterProfiler.

### 4.7. Measurement of Stomatal Aperture

A batch of the rosette leaves of the three-week-old seedlings from L9-3, L9-5, *bes1-D,* and the WT were detached and immediately used for the measurement of the stomatal aperture. Another batch of leaves from the same plants was detached and dehydrated for 1 h at room temperature. The stomatal apertures in the epidermal peels were observed under the Confocal microscope LSM800 (Carl Zeiss, Oberkochen, Germany). For each line, five leaves from ten plants were selected for the assay. Forty stomata per leaf were measured for the stomatal width and length using ImageJ software (https://imagej.nih.gov/ij/, accessed on 19 January 2021), as described by Huque et al. [56].

### 4.8. Statistical Analysis

All of the experiments were carried out in three replicates. The data are the mean ± standard deviation (SD) of three biological replicates. The significance was analyzed in the Student’s *t* tests. The * and ** represents *p* < 0.05 and *p* < 0.01, respectively.

## Data Availability

Not applicable.

## Supplementary Materials

The following supporting information can be downloaded at: https://www.mdpi.com/article/10.3390/ijms23116025/s1.
