# Supplementary material for: Maize ZmBES1/BZR1-3 and -9 Transcription Factors Negatively Regulate Drought Tolerance in Transgenic Arabidopsis
Source: Int J Mol Sci. 2022 May 27;23(11):6025. doi: 10.3390/ijms23116025 (PMC9181540; doi:10.3390/ijms23116025)

Figure S1 The KEGG enrichment analysis of DEGs in *ZmBES1/BZR1-3* expressing lines

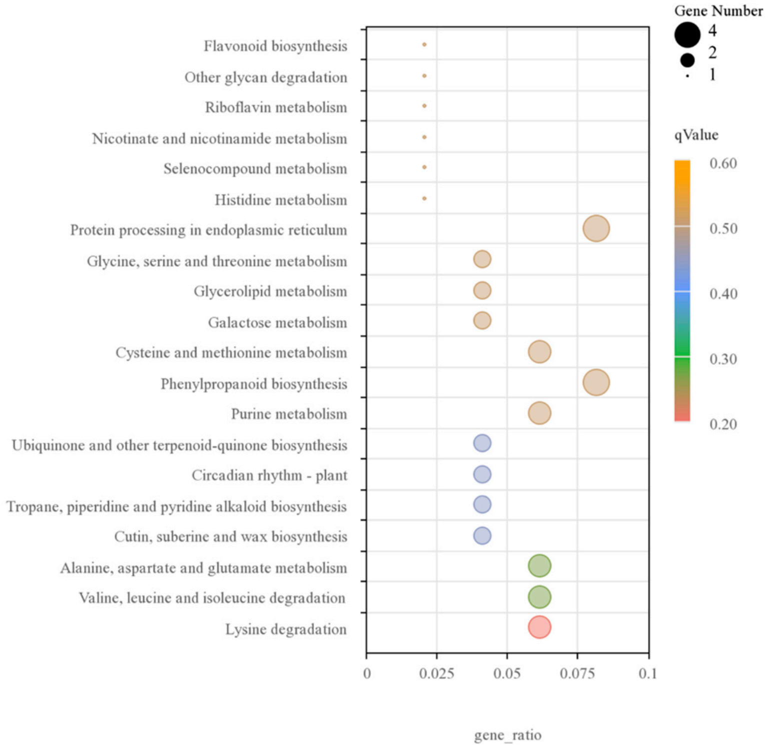

Figure S2 The KEGG enrichment analysis of DEGs in *ZmBES1/BZR1-9* expressing lines

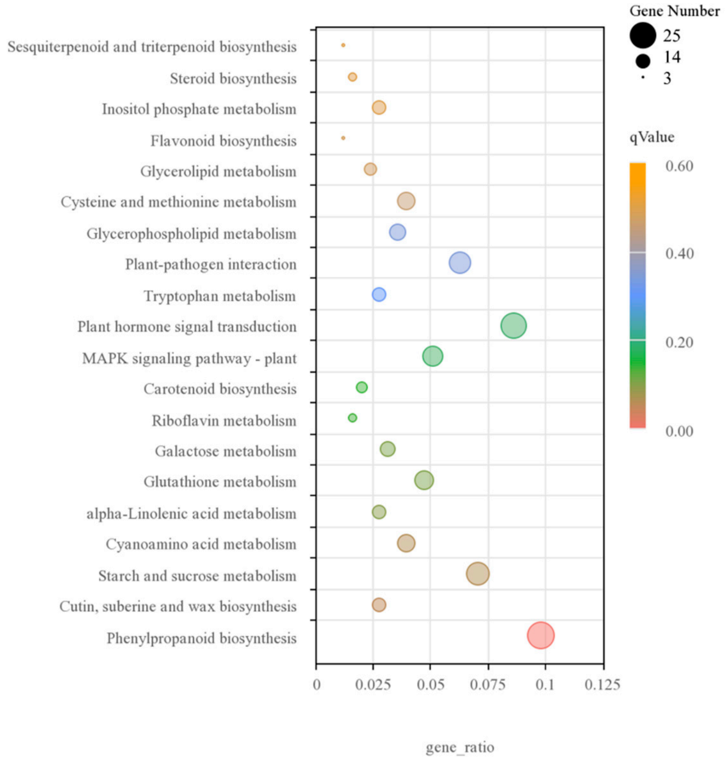

Figure S3 Multiple alignments of bHLH domain of ZmBES1/BZR1-3, ZmBES1/BZR1-9 and their homologs from *Zea mays*, *Arabidopsis thaliana*, *Oryza sativa* and *Glycine max*.

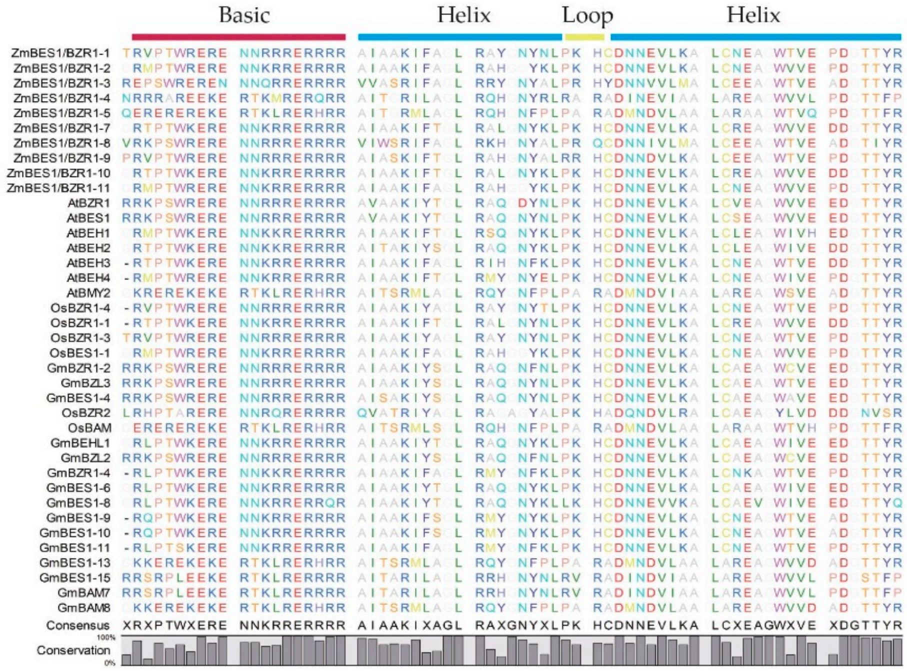

Supplement: Supplementary file 1 [file ijms-23-06025-s001.zip › Supplementary figure.pdf]
